# Supplementary material for: Penile Cancer Profile in a Central European Context: Clinical Characteristics, Prognosis, and Outcomes—Insights from a Polish Tertiary Medical Center
Source: Cancers (Basel). 2025 Jun 25;17(13):2140. doi: 10.3390/cancers17132140 (PMC12248994; doi:10.3390/cancers17132140)
Supplement: Supplementary file 1 [file cancers-17-02140-s001.zip › cancers-3615893-supplementary.pdf]

Table S1. Patient self-administrated survey.

Penile Cancer Questionnaire (Select or Write Accurate Answers)	
**Criteria**	**Patient Response**
**Age (years)**	
**Body weight (kg)**	
**Body height (cm)**	
**Body mass index (kg/m^2)**	Normal weight (BMI ≥18.5–24.9 kg/m^2)
	Overweight (BMI ≥ 25–29.9 kg/m^2)
	Obesity (≥ 30 kg/m^2)
**Size of residential area**	Rural area
	Small urban center (<20,000 inhabitants)
	Medium-sized urban center (20,000-100,000 inhabitants)
	Large metropolitan area (>100,000 inhabitants)
**Smoking**	Yes
	For how long
	Number of cigarettes per day
	No
**Circumcision status**	Circumcised
	Uncircumcised
	If circumcised—what was the reason for circumcision?
**Phimosis**	Yes
	No
**Psoriasis**	Yes
	No
**Profession**	
**Religious faith (optional)**	
**Hobbies and interests**	
